# Leveraging auxiliary measures: a deep multi-task neural network for predictive modeling in clinical research

**DOI:** 10.1186/s12911-018-0676-9

**Published:** 2018-12-12

**Authors:** Xiangrui Li, Dongxiao Zhu, Phillip Levy

**Affiliations:** 1 Department of Computer Science, Wayne State University, Detroit, MI USA; 2 Department of Emergency Medicine, Wayne State University, Detroit, MI USA; 3 Integrative Biosciences Center, Wayne State University, Detroit, MI USA

**Keywords:** Predictive modeling, Deep neural network, Auxiliary task, Multi-task learning

## Abstract

**Background:**

Accurate predictive modeling in clinical research enables effective early intervention that patients are most likely to benefit from. However, due to the complex biological nature of disease progression, capturing the highly non-linear information from low-level input features is quite challenging. This requires predictive models with high-capacity. In practice, clinical datasets are often of limited size, bringing danger of overfitting for high-capacity models. To address these two challenges, we propose a deep multi-task neural network for predictive modeling.

**Methods:**

The proposed network leverages clinical measures as auxiliary targets that are related to the primary target. The predictions for the primary and auxiliary targets are made simultaneously by the neural network. Network structure is specifically designed to capture the clinical relevance by learning a shared feature representation between the primary and auxiliary targets. We apply the proposed model in a hypertension dataset and a breast cancer dataset, where the primary tasks are to predict the left ventricular mass indexed to body surface area and the time of recurrence of breast cancer. Moreover, we analyze the weights of the proposed neural network to rank input features for model interpretability.

**Results:**

The experimental results indicate that the proposed model outperforms other different models, achieving the best predictive accuracy (mean squared error 199.76 for hypertension data, 860.62 for Wisconsin prognostic breast cancer data) with the ability to rank features according to their contributions to the targets. The ranking is supported by previous related research.

**Conclusion:**

We propose a novel effective method for clinical predictive modeling by combing the deep neural network and multi-task learning. By leveraging auxiliary measures clinically related to the primary target, our method improves the predictive accuracy. Based on featue ranking, our model is interpreted and shows consistency with previous studies on cardiovascular diseases and cancers.

## Background

Accurate prediction for disease phenotypes is one of the most important tasks in clinical research, as it can enable effective early interventions that patients are most likely to benefit. Due to the intrinsic complex biological mechanism of disease progression, successful predictive models should be capable of learning high-level information from low-level input features. However, traditional methods, such as linear regression, simplify the disease progression as additive effects of input features (i.e. age, blood pressure, renal function). Consequently, non-additive relations are not captured, potentially leading to less satisfactory predictive performances.

Deep neural networks (DNNs) have achieved great improvements for difficult predictive tasks in speech recognition, computer vision and healthcare informatics [1–4]. Compared with linear regression, DNNs have the capability of learning high-level feature representations, rendering better predictions based on those abstract features. This enables DNN to capture the non-linear relations of low-level features, making itself promising in clinical research.

Successful deep neural networks require abundant labeled data for effectively learning useful feature representations. However in clinical practice, collecting labeled data is expensive and time-consuming. As a result, only a limited amount of labeled data are available. Fitting a high-capacity model could potentially overfit the small amount of labeled data.

To avoid overfitting of DNNs, various regularization methods, such as dropout, early stopping and L2 regularization [5] have been developed. In the domain of clinic research, with defining primary targets, we can further mitigate overfitting by leveraging other clinical measures that are generated by the labeling process. As these measures are clinically related to the primary targets, we can integrate them into multi-task framework as regularization that can benefit our model.

For instance, some demographic subpopulations with hypertension are more likely to develop left ventricular hypertrophy (LVH), a form of structural heart damage that results from poor blood pressure control. Left ventricular mass indexed to body surface area (LVMI) is a commonly used method of determining when LVH is present. However, measuring LVMI requires advanced imaging but it is difficult to know which patients should undergo testing, it is challenging to predict as there is no single input features having enough explanation power for LVH. Accurately predicting LVH status for hypertension patients is critical as definitive testing to diagnose LVH, including cardiac magnetic resonance imaging (CMR), is expensive and testing every patient with hypertension would be cost prohibitive.

**Fig. 1:**
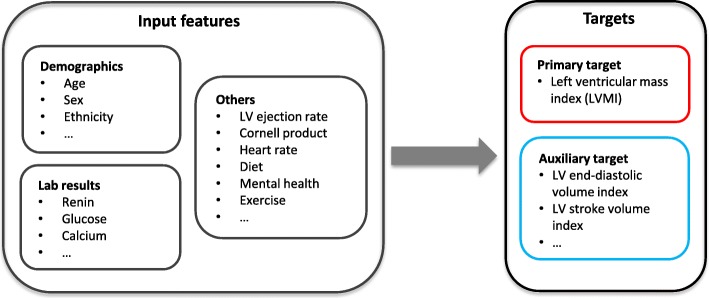
Motivating Example for GATAN. Left ventricular mass index to body surface area (LVMI) is the primary target. The labeling process also produces other measures that are clinically related to LVMI. We predict these measures as auxiliary tasks in our model

In this paper, we propose generalized auxiliary-task augmented network (GATAN), extending [6] from regression to general supervised learning tasks. GATAN is a multi-task predictive neural network that predicts the primary target and auxiliary targets simultaneously (See Fig. 1). Under the multi-task learning framework, the auxiliary tasks can be viewed as a regularization method as well as implicit data augmentation [5, 7–10]. GATAN hence can reduce the risk of overfitting. Without a universal definition of inter-task relatedness, GATAN learns task-specific feature representations, as well as a shared representation for all tasks to conceptually capture the relation. The learned representations are then combined together using a weighting mechanism; GATAN makes predictions based on the combined high-level features. Finally, to interpret GATAN, we adopt a heuristic method that analyze the learned weights to rank the contribution of input features.

## Methods

### Generalzied auxiliary-task augmented network

Taking the motivating example in Fig. 1, with LVMI being the primary target, the labeling process would produce additional CMR measures that are also characteristics of heart morphology including septal, posterior and anterior heart wall thickness. These measures are clinically related to LVMI and predictive models can exploit them as auxiliary predictive tasks.

**Fig. 2:**
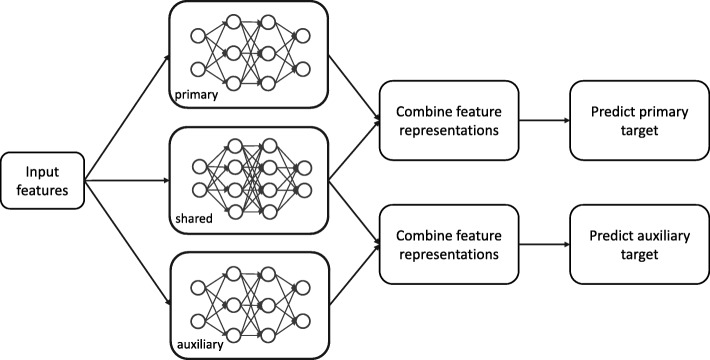
Sturcture of GATAN with one auxiliary task

However,the clinical “relevance” is not clearly defined. To circumvent this issue, GATAN models learns a feature representation that can be decomposed into a weighted sum of the shared and task-specific feature representation. The shared representation conceptually models the relevance between tasks. Figure 2 displays GATAN structure. We use feed-forward deep neural network (FDNN) [5] as the building block for GATAN.

Assume that$\left ({\vphantom {y^{c}_{a}}}\mathbf {x}, y^{c}, y^{a}\right)$ is a sample with input features **x**, primary target *y*^*c*^ and auxiliary target *y*^*a*^. The shared and task-specific feature representations are learned as follows:

1 $$ \begin{aligned} &\mathbf{h}^{s} & = f^{s}(\mathbf{x}),\\ &\mathbf{h}^{c} & = f^{c}(\mathbf{x}),\\ &\mathbf{h}^{a} & = f^{a}(\mathbf{x}),\\ \end{aligned}  $$

where *f*(·) is modeled by FDNN with multiple stacked hidden layers and non-linear activation (element-wise sigmoid action in our case). These feature representations are then combined to form the final representations **h**^*fc*^ and **h**^*fa*^:

2 $$\begin{array}{*{20}l} \mathbf{h}^{fc} &= a_{1} \mathbf{h}^{c} +a_{2} \mathbf{h}^{s}, \end{array} $$

3 $$\begin{array}{*{20}l} \mathbf{h}^{fa} &= b_{1} \mathbf{h}^{a} + b_{2} \mathbf{h}^{s}, \end{array} $$

where {*a*_1_,*a*_2_} and {*b*_1_,*b*_2_} are the weights that quantify the contributions of **h**^*s*^, **h**^*c*^ and **h**^*a*^. Note that in this formulation, **h**^*s*^, **h**^*c*^ and **h**^*a*^ are of the same dimension. As a side note, another strategy to combine the task-specific and shared feature representations is through vector concatenation **h**^*fc*^=[**h**^*c*^,**h**^*s*^]. But this approach could introduce more parameters for each **h** having enough representation power. We hence prefer the weighted sum approach when only limited amount of data is available.

To compute {*a*_1_,*a*_2_} and {*b*_1_,*b*_2_}, the *cosine*-distance “cosd” is used:

4 $$ \begin{aligned} a_{1} &= \frac{1}{2}\text{cosd}\left(\mathbf{h}^{c},\mathbf{h}^{s}\right),\\ a_{2} & = 1-a_{1}, \end{aligned}  $$

for the primary task, and

5 $$ \begin{aligned} b_{1} &= \frac{1}{2}\text{cosd}\left(\mathbf{h}^{a},\mathbf{h}^{s}\right),\\ b_{2} & = 1-b_{1}, \end{aligned}  $$

for the auxiliary task, where cosd (**v**_1_,**v**_2_)=**v**_1_·**v**_2_/(||**v**_1_||_2_||**v**_2_||_2_), ||·||_2_ is the euclidean norm of a vector. Since we use sigmoid as the activation function, {*a*_1_,*a*_2_} and {*b*_1_,*b*_2_} are positive and hence proper weights. Note that this strategy biases toward the shared feature representation and forces it to makes at least half contribution (i.e. *a*_2_,*b*_2_≥0.5) to the final feature representation for GATAN, displaying the benefits of multi-task learning.

Based on the final feature representation, the prediction $\hat {y}^{c}$ and $\hat {y}^{a}$ are calculated:

6 $$ \begin{aligned} \hat{y}^{c} & = l^{c}\left(\mathbf{W}^{c}\cdot \mathbf{h}^{fc} + h^{c}\right),\\ \hat{y}^{a} & = l^{a}\left(\mathbf{W}^{a}\cdot \mathbf{h}^{fa} + h^{a}\right), \end{aligned}  $$

where **W**^*c*^ and **W**^*a*^ are dimension-compatible vectors, *h*^*c*^ and *h*^*a*^ are bias terms, and *l*(·) is the link function depending on the specific prediction tasks. When targets are continuous, *l*(·) is the identity function; for classification tasks, *l*(·) is the sigmoid or softmax function:

$$\begin{array}{*{20}l} \sigma(x) &= \frac{1}{1+\exp(-x)}\\ \boldsymbol{\sigma}(\mathbf{x}) & = \left(\frac{\exp(x_{j})}{{\sum\nolimits}_{k=1}^{K}\exp(x_{k})}\right)_{j} (j=1,\cdots,k) \end{array} $$

The joint objective function is a sum of the loss function for each task:

7 $$ \text{minimize}_{\boldsymbol{\Theta}} \sum\limits_{i=1}^{n}L^{c}\left(y_{i}^{c},\hat{y}_{i}^{c}\right) + \omega L^{a}\left(y_{i}^{a}, \hat{y}_{i}^{a}\right),  $$

where for notational brevity, we use ***Θ*** to represent the set of parameters in the neural network, *ω* is a hyper-parameter balancing different tasks during training. We use *ω*=1 in our experiments.

For regression, the loss function is the squared loss:

$$L_{reg}\left(y^{c},\hat{y}^{c}\right) = \left(y^{c}-\hat{y}^{c}\right)^{2}. $$

For classification, it is cross-entropy:

$$L_{cla}\left(\mathbf{y}^{c},\hat{\mathbf{y}}^{c}\right) = \mathbf{y}^{c}\cdot \log \left(\mathbf{1} -\hat{\mathbf{y}}^{c}\right), $$

where we have encoded *y*^*c*^ as one-hot vector.

Note that GATAN also allows multiple auxiliary targets which can be incorporated into GATAN straightforwardly, and different types of loss functions for different tasks such as one regression task and one classification task.

### Feature ranking

Model interpretability is another important aspect in clinical practice. While there are no systematic ways to interpret deep networks, we can extend from linear regression to calculate the contribution of each input feature by back propagating each neuron’s contribution through its connections to previous layer of neurons [11].

**Fig. 3:**
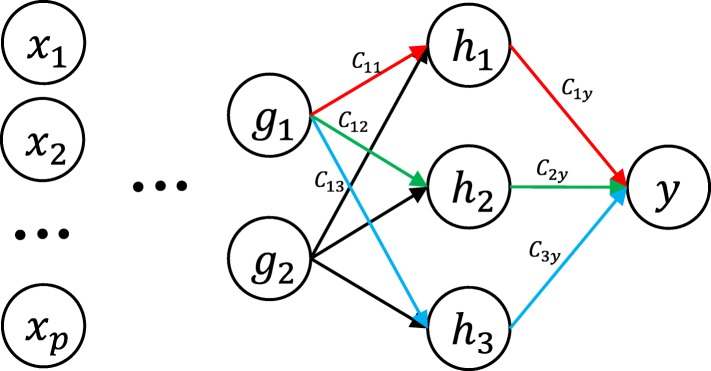
An example of calculating the contribution of hidden neurons using weight back-propagation

To see the back-propagation of each neuron’s contribution to the target, let us take an example shown in Fig. 3. Let $\mathbf {W_{1}} = \left (w_{ij}^{1}\right)_{3\times 2}$ and $\mathbf {W_{2}} = \left (w_{ij}^{2}\right)_{1\times 3}$ be the two weight matrices associated with the last two hidden layers.

*h*_*j*_’s contribution can be computed as in linear regression for {*j*:1,2,3}:

$$C_{jy} = \frac{\left|w^{2}_{1j}\right|}{\left|w^{2}_{11}\right|+ \left|w^{2}_{12}\right|+\left|w^{2}_{13}\right|}. $$

Similarly, *g*_*k*_’s (*k*=1,2) contribution *C*_*kj*_ to *h*_*j*_ is

$$C_{kj} = \frac{\left|w^{1}_{jk}\right|}{\left|w^{1}_{j1}\right|+\left|w^{1}_{j2}\right|}. $$

Then the contribution *C*_*kjy*_ from *g*_*k*_*through* *h*_*j*_ to *y* is

$$C_{kjy} = C_{kj}C_{jy}. $$

Since there are three paths from *g*_*k*_ to *y* through *h*_1_, *h*_2_ and *h*_3_, the total contribution *C*_*ky*_ of *g*_*k*_ is

$$C_{ky} = \sum\limits_{j=1}^{3}C_{kjy}. $$

We can keep propagating the contribution of neurons to input features to calculate their contributions to the target.

In GATAN, each input features can contribute to the target through the task-specific and the shared network. If $C_{ky^{c}}^{c}$ and $C_{ky^{c}}^{s}$ are the contributions of feature *x*_*k*_ through task-specific and shared network to *y*^*c*^ respectively, the overall contribution $\phantom {\dot {i}\!}C_{ky^{c}}$ for *x*_*k*_ is just the weighted sum given by

$$C_{ky^{c}} = a_{1} C_{ky^{c}}^{c} + a_{2} C_{ky^{c}}^{s},$$

which provides us a heuristic approach for interpreting GATAN, *a*_1_ and *a*_2_ are given by (4).

### Datasets and preprocessing

*Hypertension dataset* The cohort was derived from an NIH-funded study of African American patients with hypertension and elevated systolic blood pressure (>160 mm Hg) at the emergency department of Detroit Receiving Hospital. Previous studies have shown that there are disparities among hypertension patients with some who are at greater risk of LVH. This makes a DNN model that is capable of capturing complex feature interactions promising for predicting LVMI.

In the labeling process of LVMI, other measures that characterize heart morphology such as left ventricular stroke volume to body surface area (LVSVI), left ventricular end-diastolic volume indexed to body surface area (LVEDVI) and septal, posterior and anterior wall thickness, are also produced. These measures are closely relevant with LVMI and provides additional information that can be utilized in GATAN as auxiliary tasks.

**Fig. 4:**
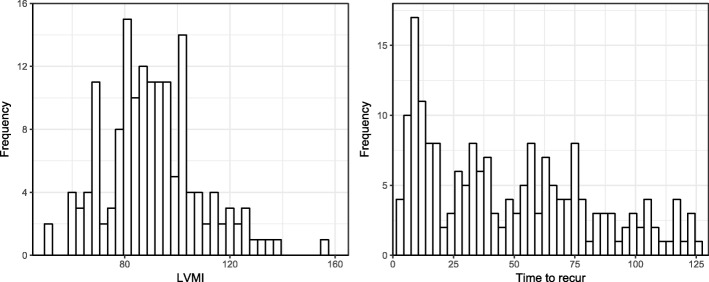
Histogram of LVMI in hypertension data (left) and time to recur for WPBC (right)

The original dataset contains 155 samples and 65 measures. These measures consists of LVMI, 59 input features (demographics, lab results, heart functioning et al.) and 5 other CMR measures as candidates of auxiliary targets. Table 1 and Fig. 4 left panel present basic statistics of targets.

**Table 1:** Descriptive statistics of LVMI and other CMR measures

	Min	1st Qtl	Median	3rd Qtl	Max	Mean
LVMI	51.06	80.06	89.72	100.83	155.66	90.81
LVSVI	9.93	22.23	28.37	33.88	53.38	28.10
LVEDVI	18.39	33.29	41.42	50.63	106.73	42.81
Septal	4.8	9.7	11.60	13.60	26.5	11.96
Posterior	2.23	9.60	11.90	14.20	22.50	12.02
Anterior	5.70	10.60	12.40	14.50	20.40	12.66

From the perspective of predictive modeling, a model only using lab results and demographics as features (34 in total) is more preferable, as they are more widely accessible and informative for disease progression, compared with the full set of features that contains heart functioning measures. Hence, we also conduct experiments with this set of features.

*Wisconsin prognostic breast cancer dataset (WPBC)* is a publicly available dataset in UCI repository [12]. The dataset contains 194 records of “time to recur” for breast cancer patients (after removing 4 cases of missing target values) and 32 features including tumor size, lymph node status and 30 measures computed from a digitized image of a fine needle aspirate (FNA) of a breast mass. These derived features include the mean value, standard error and largest/worst value for 10 features: radius, texture, perimeter, area, smoothness, compactness, concavity, concave points, symmetry and fractal dimension. The primary target is the “time to recurrence of breast cancer”; the auxiliary target is the recurrence state of being “recur” or “non-recur”.

### Implementations and evaluation metrics

First, various models in scikit-learn [13] are implemented for comparison, including non-parametric models (*k*-nearest neighbors (KNN), random forest (RF)), support vector regression (SVR), regularized linear regression based models (Ridge, Lasso and the multi-task Lasso (MTLasso)). A 4-layer perceptron (MLP-4) is also implemented whose hidden layer size is matched with GATAN.

We use Pytorch [14] for building GATAN. In our experiments, each time only one CMR measure is selected as the auxiliary target. LVEDVI is for GATAN-1 and posterior wall thickness for GATAN-2. GATAN consists of 4 layers with the dimension of hidden layers being 80 and 40. Standard gradient descent is used to train our model.

The hypertension dataset is split into training, testing and training sets by 95/35/30. For WPBC dataset, we split the data by 134/30/30. For non neural network models, 3-fold cross-validation on the training set is performed for best hyper-parameter settings. Model performances are finally reported on the testing set. We repeat this procedure 5 times.

To evaluate performance, the following three metrics are used:

- Mean squared error (MSE) measures the predictive error without considering the magnitude of target:
  $$\text{MSE} = \frac{1}{n}\left|\left|\mathbf{y}^{c} - \hat{\mathbf{y}}^{c}\right|\right|^{2}.$$
- Explained variance score (EVS):
  $$\text{EVS} = 1-\frac{\text{Var}(\mathbf{y}^{c} - \hat{\mathbf{y}}^{c})}{\text{Var}\left(\mathbf{y}^{c}\right)},$$
  where Var (·) is the variance.
- Median absolute error (MAE) is a more robust error than MSE that compute the median of absolute predictive errors:
  $$\text{MAE} = \text{Median}\left(\left|\mathbf{y}^{c} - \hat{\mathbf{y}}^{c}\right|\right).$$

Smaller MSE and MAE are better while for EVS, larger is better.

## Results and discussions

### Hypertension data

*Using entire feature set* We first experiment with the full feature set. The predictive performance on the test data is shown in Table 2. In the table, GATAN-1 and MTLasso use LVMI and LVEDVI; GATAN-2 uses posterior wall thickness as the auxiliary target.

**Table 2:** Predictive performance on hypertension dataset

Dataset	Model	KNN	RF	SVR	Ridge	Lasso	MTLasso	MLP-4	GATAN-1	GATAN-2
Full feature set	MSE	248.06	214.68	299.03	261.52	205.67	217.34	209.43	**199.76**	203.50
		(60.73)	(25.18)	(82.16)	(23.26)	(36.07)	(39.35)	(28.36)	(33.48)	(29.98)
	EVS	0.26	0.29	0.08	0.10	0.33	0.30	0.32	**0.36**	0.34
		(0.18)	(0.12)	(0.02)	(0.37)	(0.11)	(0.14)	(0.14)	(0.10)	(0.14)
	MAE	10.91	11.29	11.66	12.41	11.40	11.65	10.43	**10.20**	10.77
		(2.05)	(1.97)	(1.93)	(1.65)	(2.58)	(2.53)	(2.02)	(1.71)	(2.10)
Lab and demo	MSE	282.06	261.27	284.05	278.80	250.754	253.59	243.41	237.97	237.66
		(39.58)	(20.56)	(58.15)	(18.88)	(26.01)	(33.79)	(31.87)	(33.59)	(34.09)
	EVS	0.06	0.08	0.06	0.03	0.15	0.14	0.17	0.19	0.19
		(0.17)	(0.25)	(0.01)	(0.22)	(0.11)	(0.11)	(0.10)	(0.09)	(0.10)
	MAE	10.54	10.42	9.90	10.24	9.59	9.43	8.84	8.67	8.54
		(2.38)	(0.95)	(1.24)	(1.78)	(1.26)	(0.94)	(1.96)	(2.05)	(2.01)

The first section uses a full set of features; the second only uses lab results and demographic information

The best performance is bolded

From the table, GATAN with LVEDVI as the auxiliary target (i.e. GATAN-1) achieves the best predictive performance. For example, GATAN-1 improves MSE approximately 3% compared with Lasso; compared with MTLasso, they also performs better with margins 5% (MSE), 13% (EVS), 2% (MAE). We can also see from the table that GATAN provides performance improvements over MLP-4, due to the introduction of auxiliary tasks. This confirms that GATAN benefits from the auxiliary task in multi-task learning as a regularization.

MTLasso also introduces auxiliary tasks. However, MTLasso does not improve over Lasso. MTLasso assumes all tasks share the same subset of effective features. This is too restrictive for LVMI and LVEDVI having the same feature structure. On the contrary, GATAN has less restrictive assumption on defining the clinical “relevance”; GATAN captures the relevance by learning a shared feature representation. This implies that a proper assumption on the task relatedness is crucial for multi-task learning.

Finally, the explained variance score (EVS) is not satisfactory for all models on the testing data. From the definition, EVS is very sensitive to poor predictions. This means that all models fail for some test samples. From the histogram of LVMI (Fig. 4), we see that data might be generated from a multi-modal distribution and all models fail to capture the local data structure.

We further explored the predictive behavior of GATAN and find that models often make poor predictions at the tails of sample distribution (results no shown). For the used hypertension dataset, we find that the Pearson correlation between LVMI and calcium level is 0.79 at the right tail (LVMI >120). A two-tail correlation test shows the Pearson correlation is statistically significant (*p*-value <0.001). However, the Pearson correlation between LVMI and calcium is 0.00 for the entire dataset, -0.10 for LVMI <120. In previous studies [15, 16], it was shown that patients with LVH have strong positive correlation with serum calcium level compared to those without LVH. Our observations are consistent with these findings. This disparity of correlation between LVMI and calcium among the hypertension patients implies LVH prevalence differs among patient subgroups.

*Using demographics and lab results only* We use the same experiment setup as in the experiment with a full set of features. Table 2 shows the predictive performance with a more limited dataset. Our multi-task neural network (GATAN-1 and GATAN-2) performs better than other models, implying that our strategy of learning high-level feature representations would benefit predictive modeling. However, comparing with the setup of a full feature set, excluding heart functioning measures from the input features degrades model performances, as functional measures are expected to be more informative for predicting LVMI.

**Fig. 5:**
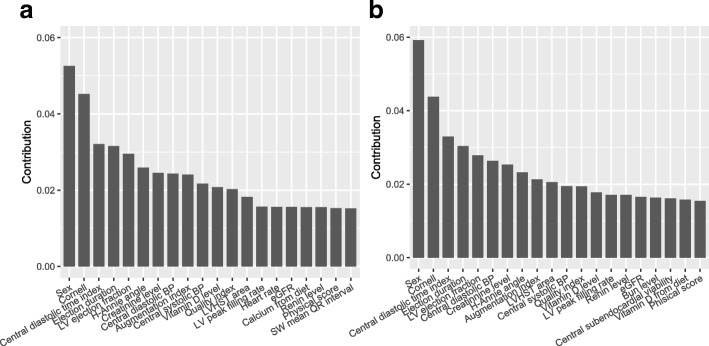
Top-20 for the complete set of features. Auxiliary target: (**a**) LVEDVI (**b**) posterior wall thickness

*Interpreting GATAN* Figure 5a and b show the top-20 features from the full set of features with respect to two different auxiliary tasks. Comparing these two figures, we see that the feature ranking in a is approximately matched with that in a. Sex is the most important feature. In the hypertension dataset, the sample mean of male versus female is 95.78 *v.s* 85.21; the difference between female and male is statistically significant with *p*-value <0.0001 for a two-sample t-test. From the figure, we also see that other features with significant contributions are functional measures, such as ejection duration, LV ejection fraction and Cornell product (an electrocardiographic predictor of LVH). This is sensible since heart structure and function are inherently related.

**Fig. 6:**
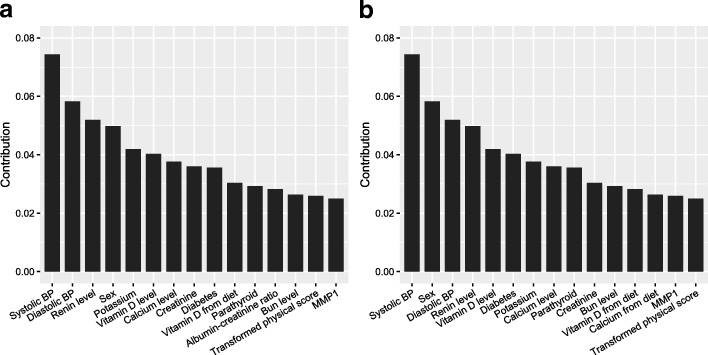
Top-15 for only lab results as features. Auxiliary target: (**a**) LVEDVI (**b**) posterior wall thickness

Figure 6 displays the top-15 features from demographics and lab results. Panel a and b are also approximately matched as those in Fig. 5. From the figure, both systolic and diastolic blood pressure are the most important features for predicting LVMI. The relationship between hypertension and LVH was the basic premise of our study. This is not surprising according to [17] that elevated blood pressure corresponds with high LVMI. Moreover, GATAN identifies more subtle relations between lab results and LVMI, including potassium, vitamin D, calcium, diabetes status, bun, renin et al. These top-ranked features accord with previous researches ([15, 18, 19]), demonstrating that feature ranking by analyzing the learned weights is a reasonable heuristic for interpreting deep neural networks.

### WPBC data

**Fig. 7:**
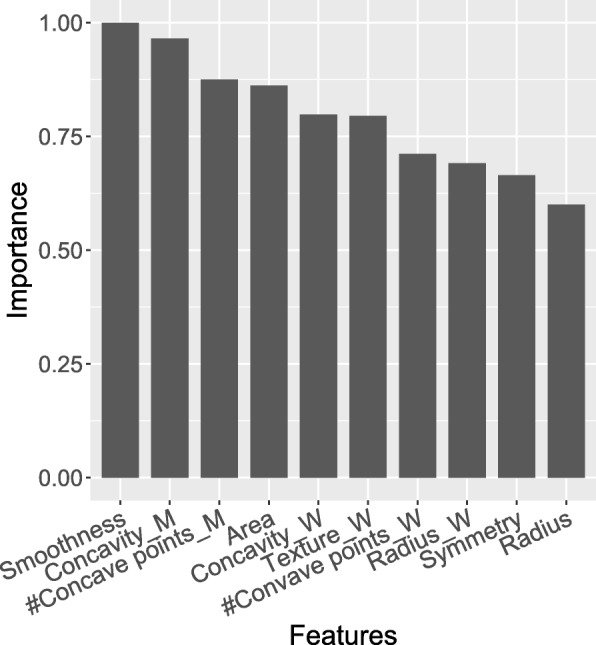
Feature contribution for WPBC dataset

Table 3 shows the performance of different models on the WPBC testing data. In terms of MSE and MAE, GATAN achieves the smallest predictive error 860.625 and 23.860 respectively. For the explained variance score (EVS), all models perform poorly. One reason accountable for this phenomenon is that the distribution of primary target “time to recur” is highly right-skewed, making it difficult for models fitting data with a long tail well. Figure 7 presents the top-10 important features for predicting “time to recur”, including FNA area, radius and texture et al. This is intuitive as morphological measures are informative about the breast cancer.

**Table 3:** Predictive performance on WPBC dataset

Dataset	Model	KNN	RF	SVR	Ridge	Lasso	MTLasso	MLP-4	GATAN
WPBC	MSE	1139.06	1189.69	1007.50	1184.38	1000.94	990.63	941.88	**860.63**
		(200.05)	(273.34)	(153.95)	(253.02)	(144.57)	(163.17)	(145.68)	(65.49)
	EVS	-0.22	-0.17	0.00	-0.21	-0.01	0.01	0.00	-0.01
		(0.15)	(0.17)	(0.01)	(0.28)	(0.15)	(0.14)	(0.01)	(0.02)
	MAE	25.48	28.00	27.16	27.78	24.58	24.16	27.09	**23.86**
		(4.94)	(3.31)	(6.08)	(4.27)	(1.32)	(3.30)	(4.88)	(0.79)

The best performance is bolded

## Conclusions

In this paper, we propose a deep multi-task neural network, GATAN, for predictive modeling in clinical research. GATAN leverages additional information in the modeling process by introducing clinical measures as auxiliary targets. As a DNN model, GATAN is capable of high-level feature learning, as well as flexibly captures the clinical relevance between the primary and auxiliary targets. As our experiments using two different datasets show, with one auxiliary task demonstrate GATAN can achieve superior performance compared with traditional models when we only have access to a limited amount of labeled data.

## Abbreviations

DNN: Deep neural network
GATAN: Generalized auxiliary-task augmented network
HTN: Hypertension
LVH: Left ventricular hypertrophy
LVMI: Left ventricular mass indexed to body surface area
